# Enhancing Hepatic Antioxidant Capacity and Carbohydrate, Lipid, and Bile Acid Metabolism in Largemouth Bass Through Dietary Betaine Supplementation

**DOI:** 10.3390/antiox15080982

**Published:** 2026-08-07

**Authors:** Jibin Lin, Manqi Yang, Qingxiang Huang, Liangliang Zhang, Liming Lu, Chunxiao Zhang, Jianchun Jiang

**Affiliations:** 1 College of Chemical Engineering, Huaqiao University, Xiamen 361021, China; 2 State Key Laboratory for Mariculture Breeding, Fisheries College of Jimei University, Xiamen 361021, China; 3 Academy of Advanced Carbon Conversion Technology, Fujian Provincial Key Laboratory of Biomass Low-Carbon Conversion, Huaqiao University, Xiamen 361021, China; 4 Fujian Besurety Biology Co., Ltd., Quanzhou 362500, China; 5 Institute of Chemical Industry of Forest Products, CAF, Nanjing 210042, China

**Keywords:** betaine, cottonseed concentrated protein, *Micropterus salmoides*, carbohydrate and lipid metabolism, bile acid metabolism, intestinal microbiota

## Abstract

This study aimed to investigate the effect of dietary betaine on the growth, glucose and lipid metabolism, and bile acid (BA) metabolism in *Micropterus salmoides* fed high-cottonseed concentrated protein (CPC) diets. In this study, *Micropterus salmoides* were fed six experimental diets: the basal diet (BD diet; high fish meal), the low-fish meal diet (LF diet; 75% of the fish meal protein in the BD diet was substituted with CPC), and four betaine-supplemented diets (1.0 g/kg, 2.0 g/kg, 4.0 g/kg, and 8.0 g/kg of betaine were added to the LF diet, designated as LFB1.0, LFB2.0, LFB4.0, and LFB8.0, respectively). After an 8-week feeding trial, compared with the BD diet, fish fed the LF diet had decreased weight gain rate, reduced feed utilization efficiency, and poor liver health and impaired metabolic function. Conversely, fish fed the LFB4.0 diet showed an increased weight gain rate and feeding rate, improved liver antioxidant capacity, and decreased hepatic lipid droplets and glycogen content. Non-targeted metabolomics of the liver revealed that betaine regulated carbohydrate and lipid metabolism and was associated with primary bile acid metabolism. Moreover, the hepatic levels of the BA regulatory factors short heterodimer partner and farnesoid-X-receptor, as well as the gene expression of BA transporter bile salt export pump, were increased in fish fed the LFB4.0 diet. Supplementing the LF diet (high CPC levels) with 4.0 g/kg betaine improves fish growth performance and enhances hepatic carbohydrate and lipid metabolism via the positive regulation of BA metabolism by betaine.

## 1. Introduction

*Micropterus salmoides*, commonly known as the largemouth bass, is a key freshwater carnivorous fish with high economic value in China [1,2]. However, the supply of high-quality protein sources such as fishmeal (FM) in traditional feeds is limited, and their prices fluctuate considerably [1,3]. This has spurred researchers to actively explore sustainable alternative protein sources. Cottonseed protein concentrate (CPC) is a promising plant-based protein source, providing benefits including high protein levels, a fairly balanced amino acid composition, and a reliable supply chain [4]. CPC holds substantial potential for extensive application in aquafeeds [5,6,7]. Recent reports indicated that CPC (replacing 50% of FM in the diet) has been used in the aquaculture of pike perch (*Sander lucioperca*) [5], common carp (*Cyprinus carpio*) [8], Chinese mitten crab (*Eriocheir sinensis*) [6], and largemouth bass [9] without compromising their growth and physiological health. However, it has been reported that high CPC diets (replacing 75% of FM in the diet) compromised growth performance and induced hepatic injury and metabolic disturbances across aquatic species. For instance, Chinese mitten crabs [6] exhibited low growth performance and reduced liver antioxidant capacity, and black carp (*Mylopharyngodon piceus*) showed dysregulated glucose and lipid metabolism [10]. Hepatitis was linked to the consumption of high levels of CPC in largemouth bass [11]. These outcomes adversely affect fish growth performance and diminish feed utilization [4]. Growing evidence indicates that excessive intake of high plant protein increased hepatic total bile acids (BAs), lipids, and collagen, upregulated BA synthesis and lipogenesis-related genes and led to hepatic steatosis and fibrosis, suggesting that diets with high-plant-protein sources may contribute to BA-mediated liver damage [12]. It was reported that high CPC diets increased hepatic triglyceride accumulation and stimulated BA synthesis while downregulating BA transport genes, and increased the levels of plasma cholic acid and hepatic chenodeoxycholic acid [13].

The liver serves as the principal site for BA synthesis, transport, and metabolism [14]. Under normal physiological conditions, BAs facilitate the digestion and absorption of intestinal lipids and play a regulatory role in glucose metabolism [15]. Moreover, BAs inhibit their own accumulation in the liver via negative feedback mechanisms, thereby maintaining the structural integrity and function of hepatocytes [15,16]. Conversely, impaired liver function may result in impaired bile excretion, causing an increase in BA concentration within the liver, subsequently inducing fibrosis, oxidative stress, and inflammation, triggering a cycle of mutual reinforcement [17]. Therefore, maintaining BA homeostasis in the liver is crucial for fish health.

Betaine is a natural zwitterionic compound with physiological functions, including regulating osmotic pressure, promoting lipid and glucose metabolism, and serving as a methyl donor [18,19]. Betaine not only exerts an attractant effect on largemouth bass [20], but also enhances the amount of carnitine in the liver and serum of fish fed high-carbohydrate diets, while reducing the deposition of hepatic glycogen and cortisol content [2]. Furthermore, dietary betaine enhanced the growth and feed intake of common carp [21] and Chinese mitten crabs [22], alleviated the accumulation of lipid droplets and the increase in triglyceride levels in the hepatopancreas of gibel carp (*Carassius auratus gibelio*) [23], and improved the antioxidant capacity of the liver of Nile tilapia (*Oreochromis niloticus*) [24]. Moreover, betaine was reported to upregulate the expression of the farnesoid X receptor (*fxr*) in liver [25]. FXR contributes significantly to the regulation of lipid and glucose metabolism and is vital for bile acid and cholesterol homeostasis [15]. However, systematic research on the potential of betaine to mitigate growth inhibition, carbohydrate and lipid metabolism disruption, and BA metabolism obstruction in largemouth bass fed a high-CPC diet is lacking.

This study focused on the role of betaine in affecting growth performance, hepatic carbohydrate and lipid metabolism, BA metabolism and intestinal microbiota in largemouth bass fed a diet high in CPC. The aim was to explore whether betaine counteracts the detrimental effects of CPC through the liver–gut–microbiota axis, thereby providing a theoretical foundation and technical parameters for the safe and efficient utilization of CPC.

## 2. Materials and Methods

### 2.1. Animal Ethics Statement

Ethical approval for all animal experiments was obtained from the Academic Ethics Center of Huaqiao University (No: A2023031) and conducted in strict accordance with applicable ethical guidelines for animal experimentation.

### 2.2. Production of Diets for Fish Farming

In this experiment, six experimental diets were prepared, including the basal diet (referred to as BD), the low-FM diet (referred to as LF, where CPC substituted 75% of the FM protein in the BD diet), and four diets with 1.0 g/kg, 2.0 g/kg, 4.0 g/kg, and 8.0 g/kg betaine (≥98%, Sigma Aldrich, St. Louis, MO, USA) added to the LF diet (LFB1.0, LFB2.0, LFB4.0, and LFB8.0) respectively. Table 1 provides the specifics of the experimental diet formulations. For the production method of the diets, refer to our previous research [26]. Briefly, the non-oily components listed in the formula table were ultra-finely pulverized and then sieved. These components were then mixed incrementally in accordance with their proportional quantities, starting from the largest to the smallest. Then, water (about 25%) was incorporated and mixed thoroughly. After that, the mixture was added to an extruder for granulation. Following the drying of the diet pellets, fish oil was uniformly sprayed on them. Finally, after cooling, the diets were stored in a low-temperature refrigerator.

### 2.3. Arrangement of the Feeding Experiment

Largemouth bass were from the same batch from a fish breeding farm in Zhangzhou, China. The fish were temporarily reared in aquaria to facilitate their adaptation to the environment in the culture room. After a period of two weeks, 450 healthy largemouth bass of uniform size (7.02 ± 0.02 g) were chosen at random and evenly distributed into 18 aquaria (corresponding to six diets, with three 120 L-aquariums for each diet). We fed the largemouth bass regularly twice a day (8:00 and 16:30) until they were satiated. Then, we removed the feces and residual diets in aquaria after each meal. Following this, we replaced approximately 20% of the water in each aquarium with fresh water. The feeding study was carried out over a period of 8 weeks. During the experiment, water conditions were controlled with dissolved oxygen levels above 6.9 mg/L, a temperature of 25 ± 2 °C, the pH at 7.1 ± 0.2, and natural lighting was provided.

### 2.4. Collection of Samples

The fish underwent a 24 h fast and then were anesthetized (eugenol, 0.01%) after the feeding experiment was completed. From each aquarium, three fish were randomly picked for whole-body composition analysis. The body weight and body length of 10 fish in each aquarium were measured. Then, blood samples were obtained from the caudal vein, using the same pretreatment method as outlined in the earlier study [27]. From each aquarium, livers of 10 fish and gallbladders of three fish were collected and rapidly preserved in liquid nitrogen. Of these livers, three livers were used for biochemical analysis and gene expression analysis, three for the analysis of bile acid profile, and four for the metabolomic analysis. Additionally, the livers of three fish per aquarium were preserved in 4% paraformaldehyde for section staining analysis. The intestinal contents and mid-intestines of three fish were collected from each aquarium for the analysis of bile acid profile and microbial community, respectively.

### 2.5. Biochemical Analysis

The methodology for analyzing the whole-body composition of the fish was referred to previous studies [27]. The detection of serum direct bilirubin (DBIL), total bilirubin (TBIL), alanine aminotransferase (ALT), aspartate aminotransferase (AST), total cholesterol (T-CHO), triglyceride (TG), and nonesterified free fatty acids (NEFAs), as well as hepatic catalase (CAT), total glutathione (T-GSH), total antioxidant capacity (T-AOC), total superoxide dismutase (T-SOD), reactive oxygen species (ROS), malondialdehyde (MDA), glycogen, pyruvate, adenosine triphosphate (ATP) and citrate synthase (CS), and total BA of gallbladder were all carried out using commercial kits (Nanjing Jiancheng Bioengineering Institute, Nanjing, China). Liver cytochrome P450 7A1, cytochrome P450 27A1, short heterodimer partner (SHP) and farnesoid X receptor (FXR) were conducted with commercial Elisa kits (Shanghai Baipeng Biotechnology Co., Ltd., Shanghai, China).

### 2.6. Histological Staining of the Liver

Following fixation, liver tissues were cut into appropriate sizes, dehydrated through a gradient of alcohol, and then embedded in paraffin. After sectioning, the sections were stained with hematoxylin (for 3 min) followed by eosin (for 1 min) successively, and then mounted. For PAS staining, a kit (G1008, Servicebio, Wuhan, China) was used, adhering strictly to the protocol of the manufacturer for the staining solution. To perform Oil Red O staining, tissues were embedded in embedding agent (OCT) and then sectioned with a cryostat microtome (CRYOSTAR NX50, Thermo Fisher, Waltham, MA, USA). For 10 min, the sections were stained using Oil Red O, followed by immersion in 60% isopropanol for differentiation (for 3 s), followed by hematoxylin staining (for 3 min). The sections were placed in 60% isopropanol for differentiation (for 3 s) after being rinsed with distilled water, soaked in the bluing solution for 1 s, rinsed in with distilled water, and then mounted. Under a microscope (DMIL LED, Leica, Wetzlar, Germany), the stained sections were viewed.

### 2.7. Analysis of Gene Expression Levels

Liver tissues were used for RNA extraction by SteadyPure Universal RNA Extraction kit (AG21017, Accurate Biology, Changsha, China). Following the quantification and quality assessment of the extracted RNA, reverse transcription was performed. For RT-qPCR analysis, the synthesized mRNA was later utilized with SYBR Green Premix *Pro Taq* HS qPCR kit (AG11718, Accurate Biology, Changsha, China). The primers for the genes were referenced from the studies on largemouth bass, and the sequences are presented in Table S1.

### 2.8. Analysis of Liver Metabolites

Liver samples were homogenized in aqueous methanol via cryogenic grinding and ultrasonic extraction, centrifuged, and the resulting supernatant was collected for further analysis. Detection was performed using LC-MS (Thermo Fisher, Waltham, MA, USA). The mass spectrometry scanning range was 70–1050 *m*/*z*, and the positive and negative ion voltages were 3500 V and −3000 V respectively. Subsequent data processing and comparison (databases: HMDB and Metlin) information on metabolites was obtained (Majorbio, Shanghai, China).

### 2.9. Analysis of the Composition of Bile Acids

Following the addition of adding the internal standard working solution and methanol extraction solution to liver, intestinal content, and serum samples respectively, the mixtures were homogenized. Subsequently, cryogenic ultrasonic treatment and centrifugation were performed. The supernatant was taken and dried, and then a 50% acetonitrile-aqueous solution was added. After mixing uniformly, cryogenic ultrasonic treatment and centrifugation were performed again, and the supernatant was taken for subsequent measurement. BA standards were mixed with methanol, and the internal standard working solution was added for measurement. The information on the 47 BA standards is shown in Table S2. Detection was carried out using LC-MS/MS (SCIEX, Framingham, MA, USA). After integrating and calibrating the data, the sample concentrations were calculated (Majorbio, Shanghai, China).

### 2.10. Analysis of Intestinal Microbial Community

Intestinal tissues were used to extract DNA, and its quality was checked prior to concentration measurement. The sequencing method remained the same as previous research (Majorbio, China) [27].

### 2.11. Statistical Analyses

The analysis used SPSS software (version 20.0) and employed general linear model procedures, with diet serving as the main effect. To compare the mean values among different treatments, Tukey’s post hoc test was applied. Data are showed as the mean ± standard error of the mean, and statistical significance was indicated by *p* < 0.05. A linear and quadratic trend analysis was also conducted [28].

## 3. Results

### 3.1. Growth Performance

Relative to the BD diet, the LF diet notably reduced the WGR and FR of fish (*p*_ANOVA_ < 0.05; Table 2). Furthermore, the WGR and FR showed a quadratic increase with the increasing addition of betaine (*p*_quadratic_ < 0.05, *p*_linear_ > 0.05; Table 2). Changing to the LFB4.0 diet reversed the increased FCR observed in fish fed the LF diet, but varying levels of betaine supplementation were not significantly influenced in a quadratic or linear way on the FCR (*p*_ANOVA_ < 0.05, *p*_quadratic_ > 0.05, *p*_linear_ > 0.05; Table 2). The diet alterations did not statistically significantly influence the HSI and CF of the fish (*p*_ANOVA_ > 0.05, *p*_quadratic_ > 0.05, *p*_linear_ > 0.05; Table 2). Fish consuming the LF diet exhibited significantly elevated VSI and AFR compared to those on the BD diet. However, feeding with the LFB4.0 diet effectively mitigated this increase, with these indices exhibiting a quadratic decrease with the increasing addition of betaine (*p*_ANOVA_ < 0.05, *p*_quadratic_ < 0.05, *p*_linear_ > 0.05; Table 2).

The analysis of the whole-body composition of fish revealed no significant variations in moisture and ash content as a result of the diet modification (*p*_ANOVA_ > 0.05, *p*_quadratic_ > 0.05, *p*_linear_ > 0.05; Table 2). However, whole-body crude protein levels were diminished in fish fed the LF diet compared to those on the BD, LFB2.0, LFB4.0, and LFB8.0 diets, while no major differences were found in the quadratic or linear alterations resulting from the added betaine (*p*_ANOVA_ < 0.05, *p*_quadratic_ > 0.05, *p*_linear_ > 0.05; Table 2). The LF diet notably raised the crude lipid content in the whole-body composition of fish compared to the BD diet, whereas there was no significant difference between fish on the LFB4.0 diet and those on the BD diet. Additionally, the crude lipid in whole-body composition of the fish exhibited a notable quadratic pattern in crude lipid content as the betaine addition level increased (*p*_ANOVA_ < 0.05, *p*_quadratic_ < 0.05, *p*_linear_ > 0.05; Table 2).

According to these results, the LFB4.0 diet was picked as the treatment group for upcoming studies to examine how betaine affects liver health in fish on the LF diet.

### 3.2. Liver Health

**Figure 1:**
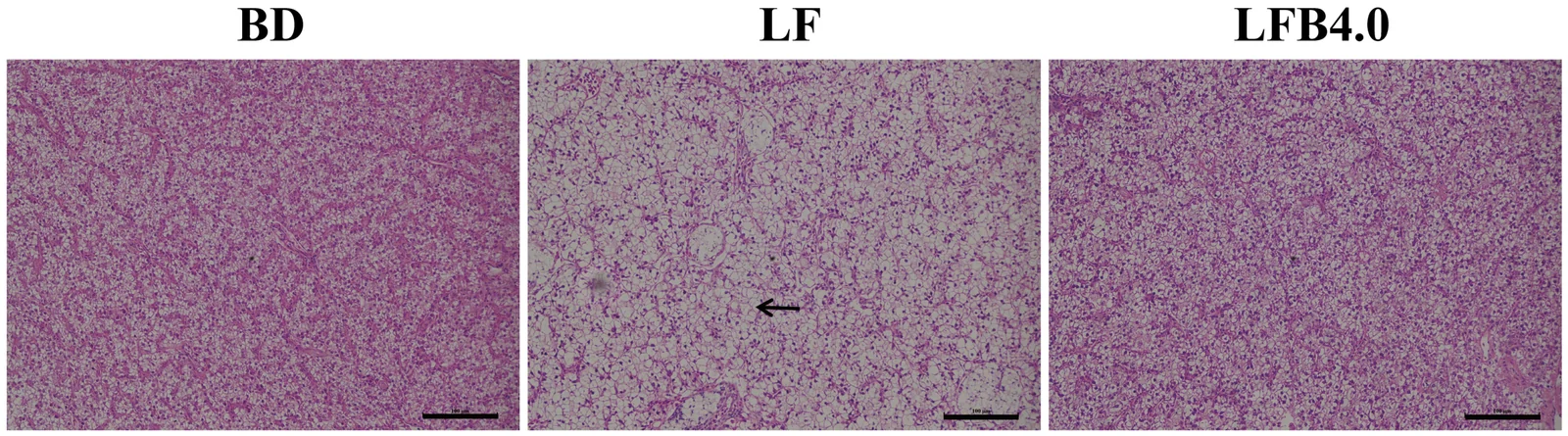
Hepatic histology H & E staining. Scale bar, 100 μm. The black arrow points to vacuolation.

The examination of liver tissue through H & E staining showed that fish fed the LF diet exhibited more pronounced liver vacuolization compared to those fed the BD and LFB4.0 diets (Figure 1). Concurrently, compared to the BD and LFB4.0 diets, the LF diet resulted in a marked increase in serum ALT and AST levels in fish (*p* < 0.05; Table 3). Among the different diet groups, serum AKP levels remained similar without significant variation (*p* > 0.05; Table 3). Fish consuming the LF diet experienced a marked reduction in hepatic CAT activity and T-GSH and T-AOC content, in contrast to those on the BD and LFB4.0 diets (*p* < 0.05; Table 3). Fish consuming the LF diet exhibited a significant reduction in hepatic T-SOD activity compared to those consuming the BD diet (*p* < 0.05; Table 3), while fish on the LF and LFB4.0 diets showed no significant differences (*p* > 0.05; Table 3). Furthermore, compared to fish fed the BD and LFB4.0 diets, fish fed the LF diet significantly increased MDA and ROS contents (*p* < 0.05; Table 3).

### 3.3. Lipid and Glucose Metabolism

**Figure 2:**
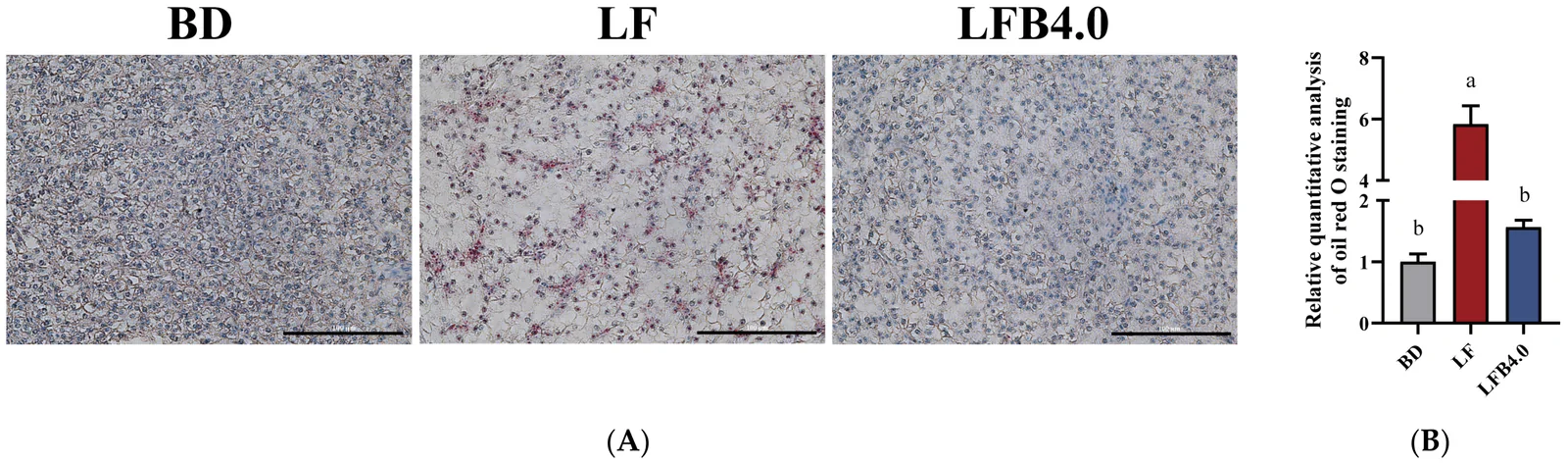
(**A**) Hepatic oil red O staining, scale bar, 100 μm. (**B**) Relative quantitative analysis of oil red O staining. Different lowercase letters on the bar chart indicate significant differences among groups (*p* < 0.05, n = 3). BD: the basic diet. LF: the low fishmeal diet. LFB4.0: adding 4.0 g/kg betaine to the LF diet.

**Figure 3:**
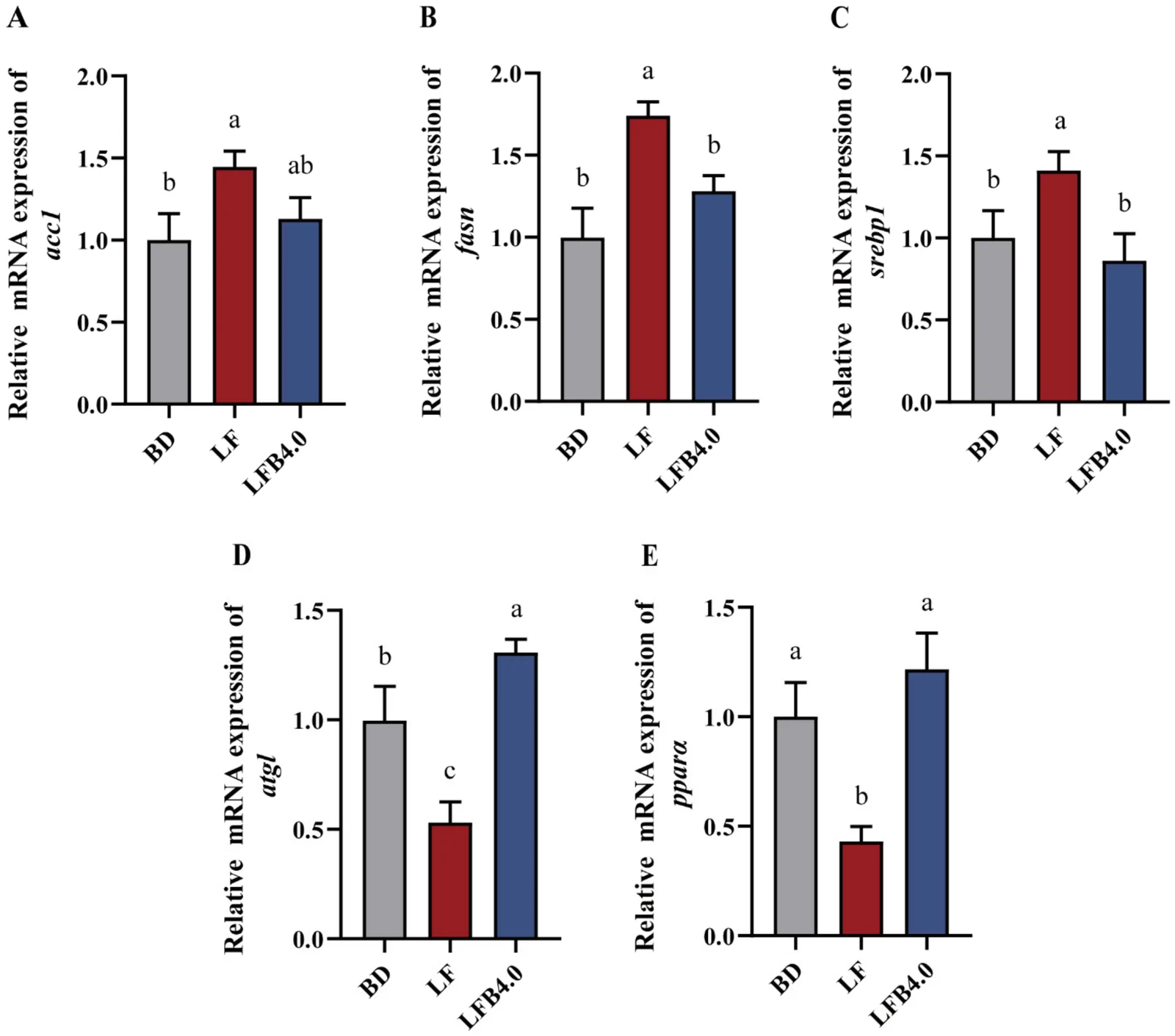
Effects of betaine on the lipid metabolism of largemouth bass. (**A**) Relative mRNA expression of acetyl-CoA carboxylase 1 (*acc1*). (**B**) Relative mRNA expression of fatty acid synthase (*fasn*). (**C**) Relative mRNA expression of sterol regulatory element-binding protein 1 (*srebp1*). (**D**) Relative mRNA expression of adipose triglyceride lipase (*atgl*). (**E**) Relative mRNA expression of peroxisome proliferator-activated receptor alpha (*pparα*). Different lowercase letters on the bar chart indicate significant differences among groups (*p* < 0.05, n = 3). BD: the basic diet. LF: the low fishmeal diet. LFB4.0: adding 4.0 g/kg betaine to the LF diet.

It was revealed through Oil Red O staining that the LF diet led to a notable increase in lipid droplets within the liver of fish compared to the BD and LFB4.0 diets (*p* < 0.05; Figure 2A,B). The LF diet significantly increased serum TG, NEFA, and T-CHO concentrations in fish when compared to the BD and LFB4.0 diets (*p* < 0.05; Table 4). Furthermore, the expression levels of lipid synthesis-related genes in the liver, including *acc1*, *fasn*, and *srebp1,* were markedly higher in fish consuming the LF diet compared to those on the BD diet, whereas the LFB4.0 diet effectively downregulated the gene expression of the *fasn* and *srebp1* (*p* < 0.05; Figure 3A–C). Compared to the BD and LFB4.0 diets, the mRNA levels of lipolysis genes (*atgl* and *pparα*) in the liver of fish on the LF diet were significantly decreased (*p* < 0.05; Figure 3D,E).

**Figure 4:**
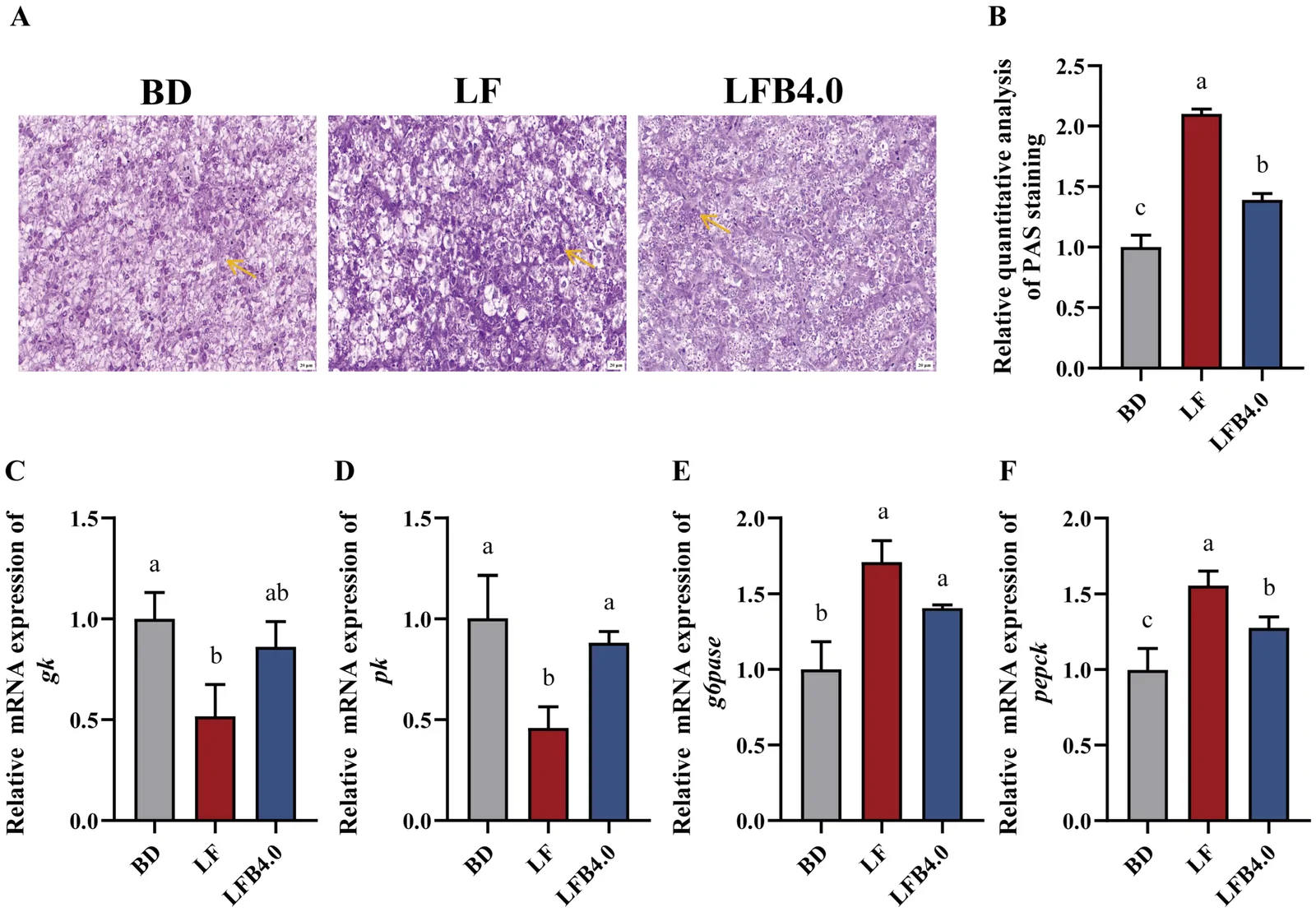
Effects of betaine on the glucose metabolism of largemouth bass. (**A**) Hepatic PAS staining, scale bar, 20 μm. The yellow arrow points to glycogen. (**B**) Relative quantitative analysis of PAS staining. (**C**) Relative mRNA expression of glucokinase (*gk*). (**D**) Relative mRNA expression of pyruvate kinase (*pk*). (**E**) Relative mRNA expression of glucose-6-phosphatase (*g6pase*). (**F**) Relative mRNA expression of phosphoenolpyruvate carboxykinase (*pepck*). Different lowercase letters on the bar chart indicate significant differences among groups (*p* < 0.05, n = 3). BD: the basic diet. LF: the low fishmeal diet. LFB4.0: adding 4.0 g/kg betaine to the LF diet.

The liver glycogen content in fish fed the LF diet was notably higher, as indicated by PAS staining and glycogen quantification, in comparison to those on the BD and LFB4.0 diets (*p* < 0.05; Figure 4A,B; Table 5). In the liver of fish fed the LF diet, the levels of pyruvate were markedly increased compared to the BD and LFB4.0 diets (*p* < 0.05; Table 5). ATP levels and the activity of CS showed the opposite trends (*p* < 0.05; Table 5). Relative to the BD diet group, the hepatic mRNA levels of the *gk* and *pk* in fish on the LF diet were significantly downregulated (*p* < 0.05; Figure 4C,D). Conversely, the mRNA levels of these genes did not significantly differ between fish consuming the LFB4.0 and BD diet (*p* > 0.05; Figure 4C,D). Compared to the BD and LFB4.0 diets, the LF diet led to a significant upregulation of mRNA levels of hepatic *g6pase* and *pepck* (*p* < 0.05; Figure 4E,F).

### 3.4. Hepatic Metabolites

**Figure 5:**
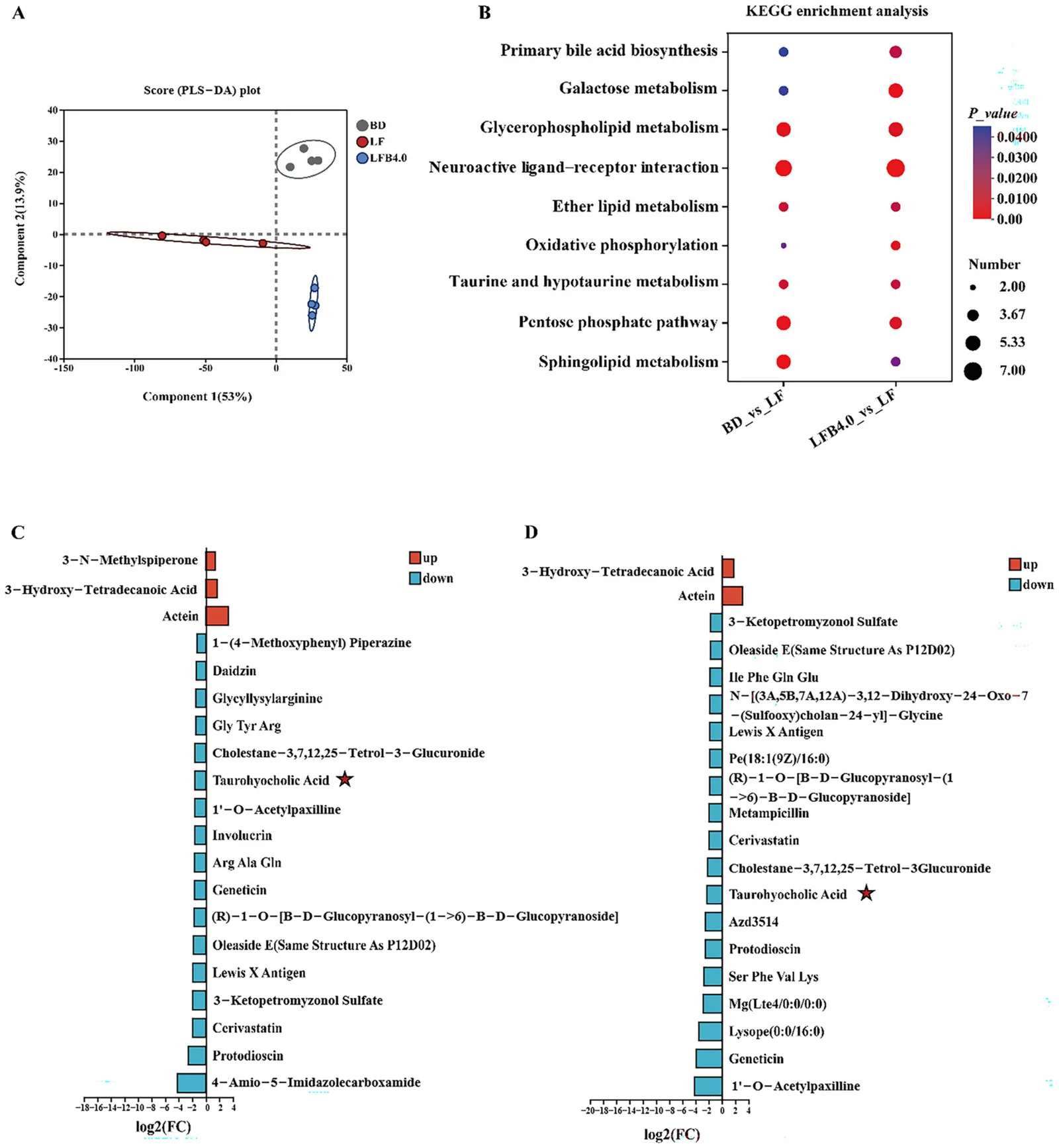
Effects of betaine on the hepatic metabolites of largemouth bass. (**A**) Partial least squares discriminant analysis (PLS-DA). (**B**) The significant KEGG pathways with an intersection in the two comparison groups (BD_vs_LF, LFB4.0_vs_LF). (**C**) The top 20 differential metabolites ranked by fold change (BD_vs_LF). The asterisk represents bile acids. (**D**) The top 20 differential metabolites ranked by fold change (LFB4.0_vs_LF). The asterisk represents bile acids. VIP ≥ 1, Fold Change = 1, *p*-value < 0.05, n = 4. BD: the basic diet. LF: the low fishmeal diet. LFB4.0: adding 4.0 g/kg betaine to the LF diet.

The samples of each experimental group did not overlap with samples of other groups (Figure 5A). Nine KEGG pathways were significantly enriched in both comparisons (BD vs. LF and LFB4.0 vs. LF), including primary bile acid biosynthesis, galactose metabolism, glycerophospholipid metabolism, neuroactive ligand–receptor interaction, ether lipid metabolism, oxidative phosphorylation, taurine and hypotaurine metabolism, pentose phosphate pathway, and sphingolipid metabolism (*p* < 0.05; Figure 5B). Among them, primary bile acid biosynthesis predominantly occurs in the liver, and the primary bile acid biosynthesis pathway can directly reflect the synthetic and metabolic functional status of the liver. In addition, within the top 20 metabolites that differ significantly in each comparison group (BD vs. LF or LFB4.0 vs. LF), it was found that the levels of taurohyocholic acid (THCA) in the BD diet group and the LFB4.0 diet group were significantly lower than those in the LF diet group (*p* < 0.05; Figure 5C,D).

### 3.5. BAs Metabolism

**Figure 6:**
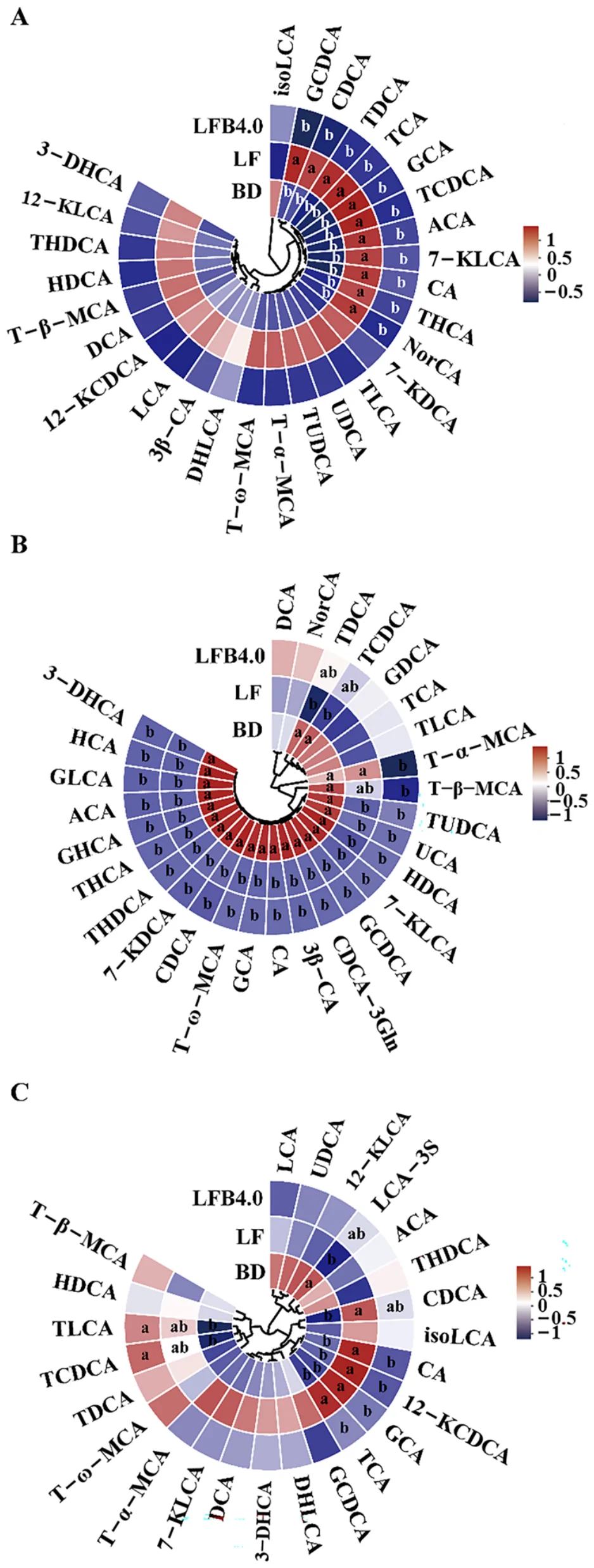
Effects of betaine on BAs metabolism of largemouth bass. (**A**) Heatmap tree of hepatic BAs. (**B**) Heatmap tree of BAs in intestinal content. (**C**) Heatmap tree of BAs in serum. Different lowercase letters on the bar chart indicate significant differences among groups (*p* < 0.05, n = 3). BD: the basic diet. LF: the low fishmeal diet. LFB4.0: adding 4.0 g/kg betaine to the LF diet.

Relative to BD and LFB4.0 groups, the LF group showed notably elevated hepatic abundances of the concentrations of glycochenodeoxycholic acid (GCDCA), taurochenodeoxycholic acid (TCDCA), taurocholic acid (TCA), glycocholic acid (GCA), chenodeoxycholic acid (CDCA), cholic acid (CA), taurohyocholic acid (THCA), and norcholic acid (NorCA) in the primary BAs pool, as well as the concentrations of taurodeoxycholate acid (TDCA), allocholic Acid (ACA), and 7-ketolithocholic acid (7-KLCA) in the secondary BAs pool (*p* < 0.05; Figure 6A). Additionally, the levels of hepatic total primary BAs and conjugated BAs in fish from the LF group were markedly more elevated than those in the BD and LFB4.0 groups (*p* < 0.05; Table 6). The BD group and the other two groups showed comparable levels of total secondary BAs (*p* > 0.05; Table 6); the levels of total secondary BAs were markedly higher in the LF group than those in the LFB4.0 group (*p* < 0.05; Table 6). Among the three dietary groups, the total unconjugated BAs levels were not significantly different (*p* > 0.05; Table 6).

The concentrations of 3-dehydrocholic acid (3-DHCA), hyocholic acid (HCA), glycohyocholic acid (GHCA), THCA, CDCA, GCA, CA, 3β-cholic acid (3β-CA), chenodeoxycholic acid-3-β-D-glucuronide (CDCA-3Gln), GCDCA, and ursocholic acid (UCA) in the primary BAs pool, as well as the concentrations of glycolithocholic acid (GLCA), ACA, taurohyodeoxycholic acid (THDCA), 7-ketodeoxycholic acid (7-KDCA), tauro-omega-muricholic acid (T-ω-MCA), 7-KLCA, hyodeoxycholic acid (HDCA), and tauroursodeoxycholic acid (TUDCA) in the secondary BAs pool, were more elevated in the intestinal content of the BD group than the LF and LFB4.0 groups (*p* < 0.05; Figure 6B). The primary BAs T-β-MCA and T-α-MCA levels were notably higher in the BD group than in the LFB4.0 group (*p* < 0.05; Figure 6B). Furthermore, higher levels of the concentrations of the primary BA TCDCA and the secondary BA TDCA were found in the BD group relative to the LF group (*p* < 0.05; Figure 6B). Among the three dietary groups, no notable changes were found in the contents of total primary and conjugated BAs (*p* > 0.05; Table 6). However, total secondary BAs and total unconjugated BAs were notably enriched in the BD group relative to the other groups (*p* < 0.05; Table 6).

In the primary BAs pool, the concentrations of CA, 12-ketochenodeoxycholicacid (12-KCDCA), GCA, and TCA were higher in the serum of fish in the LF group than those in the LF and LFB4.0 groups (*p* < 0.05; Figure 6C). The LF group exhibited notably higher serum levels of the primary bile acid CDCA compared to the BD group, while the secondary bile acid LCA-3S was reduced (*p* < 0.05; Figure 6C). In addition, the LFB4.0 group showed a significant increase in the concentrations of the secondary bile acid TLCA and the primary bile acid TCDCA compared to the BD group (*p* < 0.05; Figure 6C). In addition, the LF group displayed markedly elevated serum total primary BAs and total conjugated BAs relative to the other groups (*p* < 0.05; Table 6). The LF group had a notably higher concentration of total unconjugated BAs compared to the BD group (*p* < 0.05; Table 6). However, there were no notable variations in the concentration of total secondary BAs across the three dietary groups (*p* > 0.05; Table 6).

**Figure 7:**
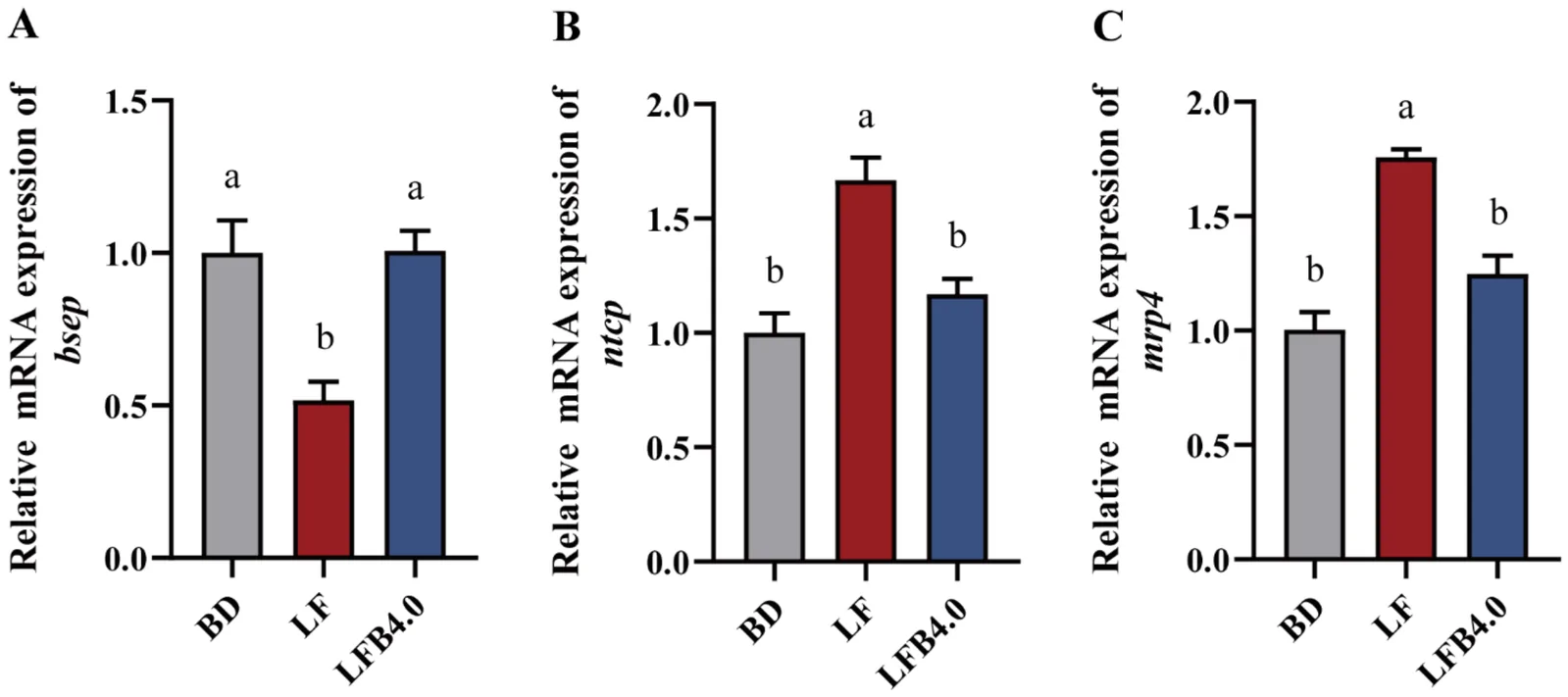
Effects of betaine on expression of genes associated with BA transport. (**A**) Relative mRNA expression of bile salt export pump (*bsep*). (**B**) Relative mRNA expression of sodium-dependent taurocholate co-transporting polypeptide (*ntcp*). (**C**) Relative mRNA expression of multidrug resistance associated protein 4 (*mrp4*). Different lowercase letters on the bar chart indicate significant differences among groups (*p* < 0.05, n = 3). BD: the basic diet. LF: the low fishmeal diet. LFB4.0: adding 4.0 g/kg betaine to the LF diet.

In contrast to fish on the BD and LFB4.0 diets, those on the LF diet exhibited a marked rise in serum total BAs, TBIL, and DBIL (*p* < 0.05; Table 6). Total BAs content in liver of fish showed the same trend as that in the serum (*p* < 0.05; Table 6). No significant intergroup variation in total BA concentration in intestinal samples was detected among the dietary treatments (*p* > 0.05; Table 6), and the trend of the content of total BAs in the gallbladder was consistent with it (*p* > 0.05; Table 6). In comparison to BD and LFB4.0 diets, the LF diet caused a significant reduction in liver FXR and SHP levels in fish (*p* < 0.05; Table 6). Conversely, the content of CYP7A1 exhibited an inverse pattern (*p* < 0.05; Table 6). Furthermore, in comparison to the BD diet, the LF diet resulted in a much higher level of hepatic CYP27A1 (*p* < 0.05; Table 6). The mRNA level of hepatic *besp* (bile salt export pump) was notably lower in fish consuming the LF diet compared to those on the BD and LFB4.0 diets (*p* < 0.05; Figure 7A), whereas the levels of *ntcp* (sodium-dependent taurocholate co-transporting polypeptide) and *mrp4* (multidrug resistance associated protein 4) expression demonstrated contrary trends (*p* < 0.05; Figure 7B,C).

### 3.6. Intestinal Microbial Community

**Figure 8:**
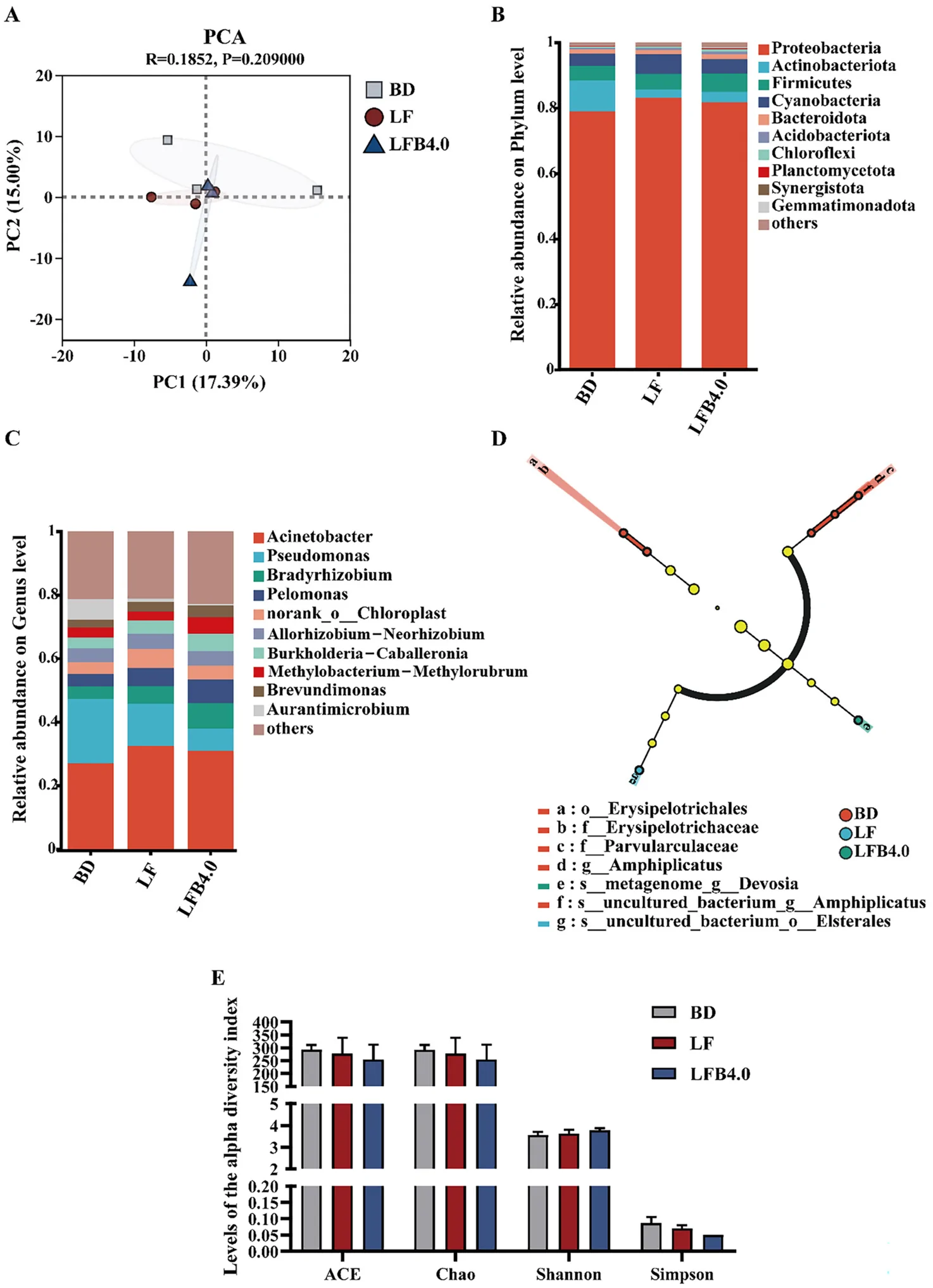
Effects of betaine on the intestinal microbial composition of largemouth bass. (**A**) Principal component analysis (PCA). (**B**) The intestinal microbial composition at the phylum level of fish in the three experimental groups. (**C**) The intestinal microbial composition at the genus level of fish in the three experimental groups. (**D**) LEfSe multi-level species difference discriminant analysis (from phylum to species). (**E**) Microbial diversity indices, *p*_ACE_ = 0.865, *p*_Chao_ = 0.866, *p*_Shannon_ = 0.598, *p*_Simpson_ = 0.220. VIP ≥ 1, Fold Change = 1, *p*-value < 0.05, n = 3. BD: the basic diet. LF: the low fishmeal diet. LFB4.0: adding 4.0 g/kg betaine to the LF diet.

Different diet groups had samples that intersected (Figure 8A). At the phylum level, the dominant intestinal microbiota of fish in the three experimental groups were Proteobacteria, Actinobacteriota, Firmicutes, Cyanobacteria, and Bacteroidota (Figure 8B). The abundance of Proteobacteria and Cyanobacteria followed the trend: BD group < LFB4.0 group < LF group (Figure 8B). Actinobacteriota was found in lower amounts in the LFB4.0 group compared to the BD group, but it was more abundant than in the LF group (Figure 8B). Firmicutes and Bacteroidota were markedly enriched in the LFB4.0 group relative to both the LF and BD groups (Figure 8B). The intestinal microbiota across the three groups was dominated at the genus level by *Acinetobacter*, *Pseudomonas*, *Bradyrhizobium*, *Pelomonas*, and norank_o__Chloroplast (Figure 8C). The abundances of *Acinetobacter* and *norank_o__Chloroplast* were higher in the LF group than in the BD and LFB4.0 groups (Figure 8C). The abundances of *Bradyrhizobium* and *Pelomonas* showed the following order: LFB4.0 group > LF group > BD group (Figure 8C). *Pseudomonas* was more abundant in the BD group than in the LF and LFB4.0 group (Figure 8C). The LEfSe analysis showed that o__Erysipelotrichales, f__Erysipelotrichaceae, f__Parvularculaceae, *g__Amphiplicatus*, and *s__uncultured_baeterium_g__Amphiplicatus* were significantly enriched in the BD group, *s__metagenome_g__Devosia* was significantly enriched in the LFB4.0 group, and *s__uncultured_bacterium_o__Elsterales* was significantly enriched in the LF group (Figure 8D). Among the experimental groups, there was no significant difference in microbial diversity indices such as ACE, Chao, Shannon, and Simpson (*p* > 0.05; Figure 8E). Additionally, the content of intestinal bile salt hydrolase (BSH) in the LF group was significantly decreased compared to that in the BD and LFB4.0 groups (*p* < 0.05; Table 6).

### 3.7. Analysis of the Correlation Between Intestinal Microbiota and Relevant Indicators of Intestinal BAs

**Figure 9:**
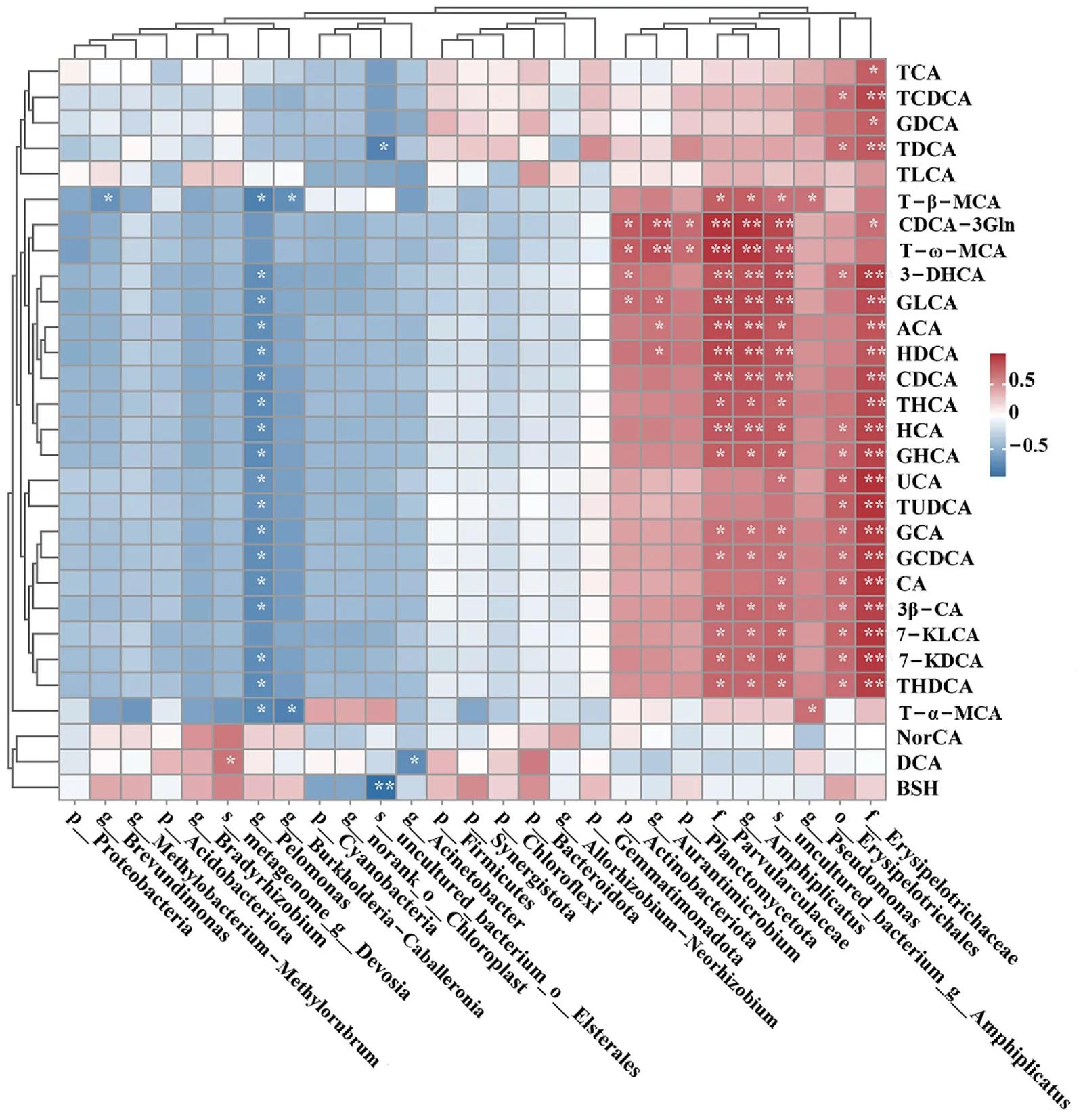
Correlation analysis between intestinal microbiota and BAs in intestinal contents, as well as the content of BSH. A notable difference is marked by an asterisk, with significance levels of * *p* < 0.05; ** *p* < 0.01.

The results of Pearson correlation analysis linking the main dominant species of intestinal microbiota and the BAs in intestinal contents and the intestinal BSH content showed that the abundance of *g__Brevundimonas* exhibited a significant negative correlation with the content of T-β-MCA (*p* < 0.05; Figure 9). A notable positive relationship was found between DCA level and the abundance of *s__metagenome_g__Devosia* (*p* < 0.05; Figure 9). The *g__Pelomonas* abundance had a significant negative correlation with the contents of T-α-MCA, THDCA, 7-KDCA, 3β-CA, CA, GCDCA, GCA, TUDCA, UCA, GHCA, HCA, THCA, CDCA, HDCA, ACA, GLCA, 3-DHCA, and T-β-MCA (*p* < 0.05; Figure 9). *g__Burkholderia-Caballeronia-Paraburkholderia* abundance exhibited a significant negative correlation with the contents of T-α-MCA and T-β-MCA (*p* < 0.05; Figure 9). *s__uncultured_bacterium_o__Elsterales* abundance showed a significant negative correlation with the content of TDCA and intestinal BSH (*p* < 0.05; Figure 9). A significant negative relationship was observed between *g__Acinetobacter* abundance and DCA content (*p* < 0.05; Figure 9). The abundance of p__Actinobacteriota demonstrated a significant positive correlation with the contents of GLCA, 3-DHCA, CDCA-3Gln, and T-ω-MCA (*p* < 0.05; Figure 9). The *g__Aurantimicrobium* had a significant positive correlation with the contents of HDCA, ACA, GLCA, T-ω-MCA, and CDCA-3Gln (*p* < 0.05; Figure 9). Significant positive correlations were found between the abundance of p__Planctomycetota and the levels of CDCA-3Gln, and T-ω-MCA (*p* < 0.05; Figure 9). The abundances of f__Parvularculaceae, *g__Amphiplicatus*, and *s__uncultured_bacterium_g__Amphiplicatus* were significantly positively correlated with the contents of THDCA, 7-KDCA, 7-KLCA, 3β-CA, GCDCA, GCA, GHCA, HCA, THCA, CDCA, HDCA, ACA, GLCA, 3-DHCA, T-ω-MCA, CDCA-3Gln, and T-β-MCA (*p* < 0.05; Figure 9). The abundance of *s__uncultured_bacterium_g__Amphiplicatus* showed a significant positive correlation with the contents of CA and UCA (*p* < 0.05; Figure 9). *g__Pseudomonas* abundance was significantly positively correlated with the contents of T-β-MCA and T-α-MCA (*p* < 0.05; Figure 9). The o__Erysipelotrichales abundance was significantly positively correlated with the contents of THDCA, 7-KDCA, 7-KLCA, 3β-CA, CA, GCDCA, GCA, TUDCA, UCA, GHCA, HCA, 3-DHCA, TDCA, and TCDCA (*p* < 0.05; Figure 9). The f__Erysipelotrichaceae abundance was significantly positively correlated with the contents of THDCA, 7-KDCA, 7-KLCA, 3β-CA, CA, GCDCA, GCA, TUDCA, UCA, GHCA, HCA, THCA, CDCA, HDCA, ACA, GLCA, 3-DHCA, CDCA-3Gln, TDCA, GDCA, TCDCA, and TCA (*p* < 0.05; Figure 9).

### 3.8. Correlation Analysis Between Hepatic BA-Related Genes and Related Indicators

**Figure 10:**
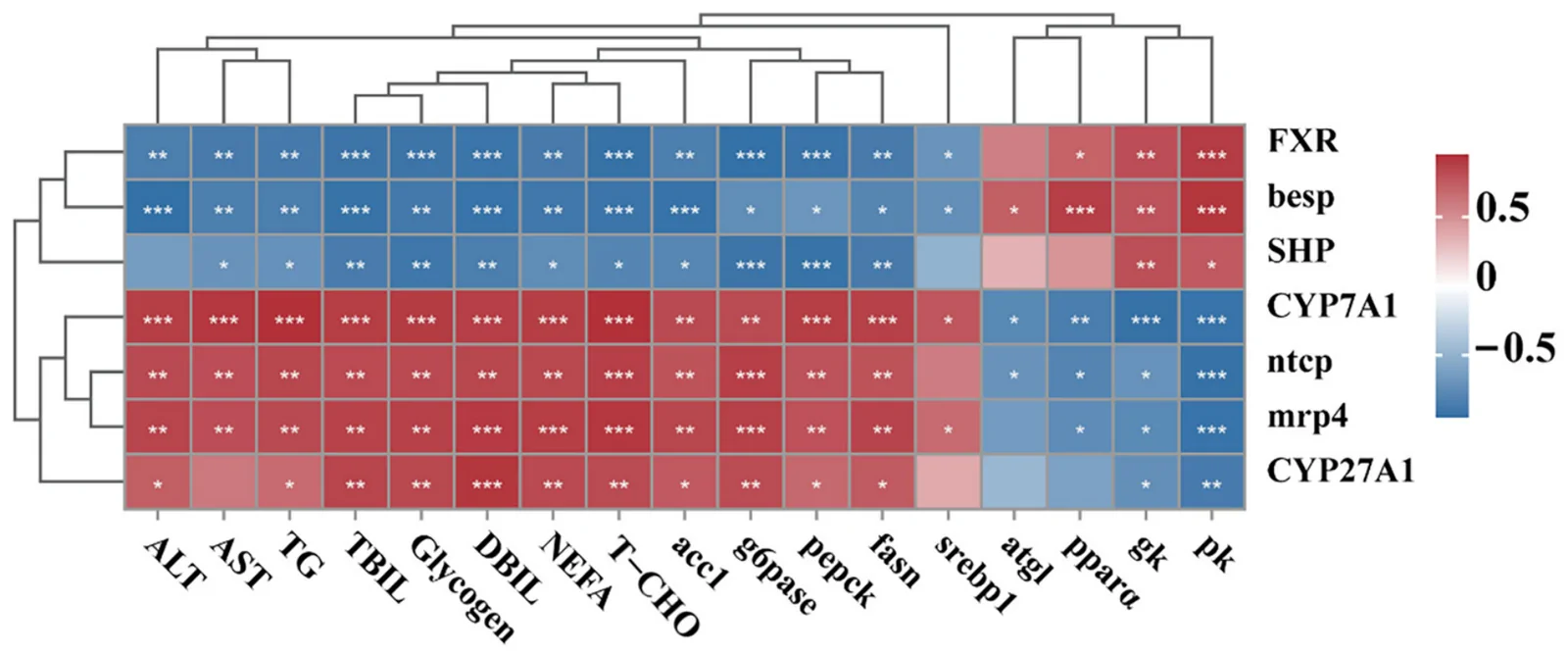
Correlation analysis between hepatic BA-related genes (genes related to the regulation and transport of BAs) and biochemical indicators. A notable difference is marked by an asterisk, with significance levels of * *p* < 0.05; ** *p* < 0.01; *** *p* < 0.001.

The hepatic FXR content was strongly positively correlated with the hepatic gene expression levels of *pk*, *gk*, and *pparα*. An inverse correlation was observed between the hepatic mRNA levels of *srebp1*, *fasn*, *pepck*, *g6pase*, and *acc1*, and the contents of T-CHO, NEFA, DBIL, TBIL, TG, AST, and ALT in the serum and the level hepatic glycogen (*p* < 0.05; Figure 10). A marked positive association was detected between the hepatic mRNA level of the *besp* and mRNA level of *pk*, *gk*, *pparα*, and *atgl*, but significantly negatively correlated with the contents of T-CHO, NEFA, DBIL, TBIL, TG, AST, and ALT in the serum, as well as the levels of hepatic glycogen and the hepatic gene expression levels of *srebp1*, *fasn*, *pepck*, *g6pase*, and *acc1* (*p* < 0.05; Figure 10). The contents of CYP7A1 and CYP27A1 in the liver and the hepatic mRNA levels of the *ntcp* and *mrp4* were all significantly negatively correlated with the hepatic mRNA levels of *pk* and *gk*, but significantly positively related to the contents of T-CHO, NEFA, DBIL, TBIL, TG, and ALT in the serum, the hepatic gene expression levels of *fasn*, *pepck*, *g6pase*, and *acc1*, and hepatic glycogen content (*p* < 0.05; Figure 10). In addition, the content of CYP7A1 in the liver was also significantly inversely related to the hepatic mRNA levels of *pparα* and *atgl*, and a significant positive correlation was observed with the hepatic mRNA level of *srebp1*, as well as the content of serum AST (*p* < 0.05; Figure 10). Hepatic mRNA expression level of *ntcp* was negatively associated with both *pparα* and *atgl*, but positively associated with serum AST concentrations (*p* < 0.05; Figure 10). There was a significant inverse association between the expression level of hepatic *mrp4* and *pparα*, whereas a significant direct association was observed with the mRNA level of srebp1 and serum AST content (*p* < 0.05; Figure 10).

## 4. Discussion

The experimental findings demonstrated that the LF diet significantly reduced both the WGR and FR in fish. These results align with previous studies on largemouth bass, reporting that the intake of high-dose CPC results in a reduction in FR that might lead to poor growth [1,13,29]. The adverse effects induced by CPC were similarly seen in shrimp (*Litopenaeus vannamei*) [30], hybrid culter [7], and rainbow trout (*Oncorhynchus mykiss*) [31]. Furthermore, a report on hybrid culter demonstrated that high-dose CPC increased crude lipid content [7], which was supported by the present results. The propensity of carbohydrate-rich diets to trigger abdominal fat deposition in carnivorous species may underlie this observed lipid deposition [32,33], which also accounts for the observed significant increase in the VSI and AFR of fish fed the LF diet in the study. However, the present study demonstrated that betaine supplemented at 4 g/kg in the LF diet significantly alleviated these adverse effects associated with the LF diet on fish. The capacity of betaine to boost feeding behavior is likely responsible for this effect [20,22,34] and the positive modulation of glucose and lipid metabolism [34,35].

Largemouth bass fed high-dose CPC diets have been reported to show abnormal histological structures in the liver and increased serum ALT and AST levels [13]. The results of this study are consistent with these findings, indicating that fish fed the LF diet suffered hepatic damage [4]. Additionally, this study revealed reduced hepatic antioxidant defense capacity in fish fed the LF diet. Consistent with previous studies on largemouth bass and hybrid culter, excessive CPC intake has been associated with increased hepatic MDA content [7,11]. Notably, the LFB4.0 diet in this study ameliorated these hepatic lesions. Similarly, the promoting effect of betaine on the ability of the liver to maintain redox homeostasis was demonstrated in Chinese mitten crab [22] and Caspian trout (*Salmo trutta*) [36]. This advancement is essential for upholding the liver’s structural integrity and facilitating the normal function of the liver, with a focus on carbohydrate and lipid metabolism regulation [37].

The findings indicated that the LF diet contributed to hepatic lipid buildup, accompanied by upregulated lipid synthesis genes and downregulated lipolysis-related genes. These results are consistent with reports of excessive CPC intake inducing an increase in hepatic TG and T-CHO contents in largemouth bass [13], as well as increased concentrations of these two indicators in the serum of black carp [10]. In contrast, fish fed the LFB4.0 diet did not exhibit this lipid metabolism disorder, suggesting that betaine favorably impacts lipid metabolism. Studies on various fish species have demonstrated the beneficial effects of betaine, reducing the hepatic oil-red staining area of *M. amblycephala* fed a high-carbohydrate diet [38], decreasing the serum concentrations of T-CHO and TG in Nile tilapia [39], and downregulating the mRNA levels of genes associated with lipid synthesis (*srebp1*, *acc1*, and *fasn*) in the liver of mandarin fish [34].

Reports have demonstrated the adverse impact of an increased proportion of plant-based protein in the diets of carnivorous fish [40,41,42], and this is linked to the limited tolerance of most carnivorous fish to carbohydrates [43]. The CPC diet contained significantly more carbohydrates than the FM diet [13]. Moreover, in carnivorous fish, a high-carbohydrate diet not only leads to abnormal lipid metabolism but also disturbs normal glucose metabolism [33,44]. These effects were further verified in this research. The fish fed the LF diet in this study exhibited increased hepatic glycogen and pyruvate levels, whereas the ATP content and CS activity decreased. This suggests inhibition of the tricarboxylic acid cycle, with excess pyruvate potentially redirected towards fatty acid synthesis instead of mitochondrial oxidation [45]. In contrast, the fish fed the LFB4.0 diet showed the opposite state. Betaine has been reported to decrease the content of hepatic glycogen and enhance the utilization of glucose by promoting glycolysis, thereby enhancing the glucose metabolism irregularities in largemouth bass fed a carbohydrate-heavy diet [2]. Similar results were observed in this study: betaine supplementation was associated with reduced hepatic lipid accumulation and glycogen deposition, downregulation of lipogenic genes (*fasn* and *srebp1*), upregulation of lipolytic genes (*atgl* and *ppara*), and recovery of glycolytic gene expression (*pk*). These effects suggest that betaine improved glucose utilization and reduced the diversion of excess pyruvate toward lipid synthesis.

To further investigate the mechanism by which betaine regulates carbohydrate and lipid metabolism, this study performed hepatic metabolomics analysis. The current study showed that the primary BA metabolism pathway, which is associated with carbohydrate and lipid metabolism [15], exhibited significant differences in both experimental comparison groups (BD vs. LF, and LFB4.0 vs. LF). An appropriate level of hepatic BAs is beneficial for enhancing the digestion and absorption of lipids, regulating the synthesis and metabolism of BAs, modulating glucose metabolism and maintaining liver health [46]. Cholestasis results in the reflux of BAs into the bloodstream, leading to a corresponding increase in the levels of serum bilirubin [47]. Consequently, animals experiencing cholestasis typically exhibit higher serum levels of AST, ALT, DBIL, TBIL, and total BAs [47,48]. Importantly, the LF group had notably higher levels of total BAs in serum and liver, as well as DBIL and TBIL in serum relative to both the BD and LFB4.0 groups, indicating that the LF diet may cause cholestasis in fish. Previous studies on rainbow trout demonstrated that a high-carbohydrate diet upregulated BA synthesis in the liver, even exceeding the level required to compensate for the fecal excretion of BAs [49]. Furthermore, excessive CPC intake had been reported to promote BAs synthesis in largemouth bass [13], reinforcing the results of this study.

BAs are categorized as either primary or secondary based on the production process [15]. In the liver, primary BAs are produced from cholesterol via a series of enzyme-driven reactions, with CYP7A1 and CYP27A1 playing key roles in the initial synthesis phase [50]. The study revealed that hepatic CYP7A1 levels were notably elevated in the LF group relative to the other groups, suggesting that the LF diet enhances the capacity of the fish liver for BAs synthesis. Some BAs, such as CDCA and CA, activate *fxr* to induce *shp*, subsequently inhibiting *cyp7a1* transcription in hepatocytes to prevent cholestasis caused by BA overproduction [51]. The study demonstrated that while fish fed the LF diet had much higher levels of hepatic total BAs, CDCA, and CA than those fed other diets, their levels of hepatic FXR and SHP were significantly decreased. This observation aligns with previous research on turbot (*Scophthalmus maximus*) and largemouth bass. Exposure to a high-glucose medium downregulated *shp* and *fxr* expression in turbot primary hepatocytes [52]. Similarly, excessive CPC consumption decreased *fxr* and *shp* expression in the intestines of largemouth bass [13]. This may explain the elevated hepatic CYP7A1 levels in fish fed the LF diet in this research. Furthermore, diminished transcription of BA transport-related genes has been reported under high-carbohydrate dietary conditions [13,40]; the results of this study confirm that *bsep* codes for the bile salt export pump located on the canalicular membrane, and FXR induces *bsep* expression to maintain BA homeostasis in hepatocytes and prevent cholestatic liver injury [53]. *bsep* expression was notably downregulated in the LF group relative to the other groups, indicating impaired BA excretion from the liver into the biliary tract, which may directly contribute to cholestasis [47]. This impairment may also account for the increased content of hepatic BAs in the LF group. Furthermore, during cholestasis, *fxr* induces *mrp4* as an adaptive mechanism to facilitate BA excretion into the bloodstream [53], which explains the increased levels of serum total BAs in fish consuming the LF diet. These findings suggest that betaine plays a role in maintaining BAs metabolism homeostasis in the liver of fish fed a high-CPC diet.

The function of the BA pool is determined not only by its size but also by the types of BAs [14]. BAs are classified as either hydrophilic or hydrophobic, according to their structure. Hydrophobic BAs exhibit a certain degree of toxicity, which is positively correlated with their hydrophobic nature [54]. BAs conjugation with amino acids enhances their water solubility [55]. In the liver, the content of total conjugated BAs in fish fed the LF diet was higher than that of fish fed the BD and LFB4.0 diets, and the contents of hydrophobic BAs, such as CDCA and CA, remained significantly higher in the liver of fish fed the LF diet relative to the other two groups. Meanwhile, the serum levels of total unconjugated BAs and CDCA in the LF diet group were significantly higher than those in the BD diet group, and the level of serum CA was significantly higher than of fish in the BD and LFB4.0 diet groups. These findings align with studies on largemouth bass reporting that substituting FM with CPC notably increased plasma CA levels and hepatic CDCA levels [13]. This indicates that the LFB4.0 diet positively influences the optimization of BA composition in the hepatic and serum BA pools.

Primary BAs are produced from cholesterol in the liver, whereas secondary BAs are formed due to microbial enzyme activity [15]. Microbes convert primary BAs into secondary BAs through 7α-dehydroxylation, 7β-epimerization, and deconjugation reactions [15]. Different intestinal microbiota can secrete different metabolic enzymes to mediate BA transformation [17]. BSH, primarily produced by gut microbes, acts as a rate-limiting enzyme during secondary BA production by catalyzing the deconjugation reaction of conjugated BAs, increasing the amount of free BAs available for further modification or absorption by intestinal bacteria [50]. However, the study demonstrated that fish fed the LF diet had lower intestinal BSH levels than the other diets. Furthermore, fish fed high-carbohydrate diets exhibit reduced gallbladder contraction, potentially resulting in lower BAs concentrations in the intestinal contents [49]. Thus, the increased BSH level facilitates secondary BAs synthesis [15], which may contribute to the observed increase in the content of total unconjugated BAs within the intestinal contents of fish consuming the BD diet. Furthermore, there was a significant negative correlation between the abundance of *s__uncultured_bacterium_o__Elsterales*, an intestinal biomarker bacterium of fish consuming the LF diet, and the content of intestinal BSH. However, no studies have reported whether this bacterium secretes BSH. In the LFB4.0 diet group, several intestinal bacteria with higher levels of abundance, including *g_Brevundimonas*, *g__Pelomonas*, and *g__Burkholderia-Caballeronia-Paraburkholderia*, were all significantly negatively correlated with T-β-MCA, which was lower in the intestinal contents of fish in the LFB4.0 diet group. It was reported that T-β-MCA inhibited *fxr* expression [15], whereas betaine activated *fxr* expression [25], which is beneficial for regulating the BA pool size.

## 5. Conclusions

This study demonstrated that the LF diet (high-CPC diet) negatively impacted the growth performance and liver function of largemouth bass. Conversely, supplementation of the LF diet with 4.0 g/kg of betaine increased the WGR and FR, enhanced hepatic antioxidant capacity, and sustained normal hepatic carbohydrate, lipid, and BA metabolism.

## Figures and Tables

**Table 1:** Formulation of the basic diet and proximate composition of experimental diets (dry matter, g/kg).

Ingredients	Experimental Diets					
	BD	LF	LFB1.0	LFB2.0	LFB4.0	LFB8.0
Fish meal	500.00	125.00	125.00	125.00	125.00	125.00
Corn starch	90.00	90.00	90.00	90.00	90.00	90.00
Soybean meal	80.00	80.00	80.00	80.00	80.00	80.00
Casein	90.00	90.00	90.00	90.00	90.00	90.00
Cottonseed protein concentrate	0.00	398.00	398.00	398.00	398.00	398.00
Betaine	0.00	0.00	1.00	2.00	4.00	8.00
Ca(H_2_PO_4_)_2_	10.00	10.00	10.00	10.00	10.00	10.00
Vitamin mixture ^1^	4.00	4.00	4.00	4.00	4.00	4.00
Mineral premix ^2^	6.00	6.00	6.00	6.00	6.00	6.00
L-ascorbate-2-phosphate	0.20	0.20	0.20	0.20	0.20	0.20
Microcrystalline cellulose	164.80	121.80	120.80	119.80	117.80	113.80
Choline chloride	5.00	5.00	5.00	5.00	5.00	5.00
Fish oil	50.00	70.00	70.00	70.00	70.00	70.00
Proximate compositions (%)						
Crude protein	50.05	50.12	50.10	50.06	50.02	50.13
Crude lipid	10.12	10.09	10.02	10.07	10.10	10.09
Moisture	5.88	6.03	6.08	6.11	6.09	6.03
Nitrogen free extract	8.83	14.31	14.01	13.96	14.13	14.66

^1,2^ Mineral premix and vitamin mixture have been manufactured, referred to previous studies [27].

**Table 2:** Effect of betaine on growth performance and the whole-body composition of largemouth bass.

Parameters	Diets						Pooled SEM	*p*-Value		
	BD	LF	LFB1.0	LFB2.0	LFB4.0	LFB8.0		ANOVA	Linear	Quadratic
Growth performance										
WGR (%)	463.30 a	337.15 e	350.75 de	392.07 cd	436.81 ab	405.21 bc	8.70	<0.001	0.836	0.047
FR (%)	2.52 a	2.39 b	2.39 b	2.47 ab	2.54 a	2.49 a	0.02	<0.001	0.197	0.037
FI (g/fish)	32.67 a	25.28 d	26.07 cd	28.76 bc	31.72 ab	29.68 ab	0.59	<0.001	0.633	0.055
FCR	1.01 c	1.07 a	1.05 ab	1.04 ab	1.03 bc	1.04 ab	0.01	<0.001	0.451	0.116
HSI (%)	1.82	1.90	1.90	1.91	1.84	1.86	0.02	0.060	0.937	0.052
VSI (%)	7.83 c	8.10 a	8.11 a	8.04 ab	7.94 bc	8.05 ab	0.02	<0.001	0.333	0.042
CF (g/cm^3^)	1.98	1.90	1.90	1.93	1.98	1.94	0.02	0.080	0.820	0.270
AFR (%)	1.64 c	1.86 a	1.83 ab	1.81 ab	1.73 bc	1.79 ab	0.02	<0.001	0.377	0.031
Whole-body composition, (%)										
Moisture	76.59	76.13	76.63	76.15	75.09	74.43	1.25	0.802	0.158	0.306
Crude protein	19.66 ab	16.84 c	17.15 bc	19.82 a	21.22 a	19.49 ab	0.52	0.001	0.080	0.125
Crude lipid	7.05 b	7.60 a	7.54 a	7.55 a	7.28 ab	7.45 a	0.07	0.002	0.354	0.033
Ash	3.54	3.49	3.36	3.62	3.36	3.64	0.15	0.739	0.785	0.713

Note: WGR: weight gain rate. FR: feeding rate. FI: feed intake. FCR: feed conversion ratio. HSI: hepatosomatic index. VSI: viscerosomatic index. CF: condition factor. AFR: abdominal fat rate. Data in same row with different letters indicates significant differences (*p* < 0.05, n = 3). WGR (%) = 100 × (final body weight-initial body weight)/initial body weight. FR (%/d) = 100 × feed intake/[(initial body weight + final body weight)/2]/feeding days. FI (g/fish) = feed intake/fish number. FCR = feed consumption/weight gain. HSI (%) = 100 × liver weight/final body weight. VSI (%) = 100 × viscera weight/final body weight. CF (g/cm^3^) = 100 × final body weight/final body length^3^. AFP (%) = (individual abdominal fat weight/individual body weight) × 100.

**Table 3:** Effect of betaine on liver health of largemouth bass.

Parameters	Diets			Pooled SEM	*p*-Value
	BD	LF	LFB4.0		ANOVA
Serum					
ALT (U/L)	2.69 b	4.54 a	2.50 b	0.13	<0.001
AST (U/L)	24.17 b	34.77 a	24.03 b	1.05	0.001
AKP (U/L)	49.12	54.67	49.15	1.83	0.129
Liver					
CAT (U/mg prot)	2.66 a	1.68 b	2.49 a	0.07	0.001
T-GSH (μmol/mg prot)	199.28 a	92.83 c	157.18 b	6.49	<0.001
T-AOC (mmol/g)	1.09 a	0.99 b	1.07 a	0.01	<0.001
T-SOD (U/mg prot)	186.53 a	144.49 b	167.22 ab	6.92	0.020
MDA (nmol/mg prot)	1.46 b	5.02 a	1.49 b	0.18	<0.001
ROS (Arbitrary Unit/mg prot)	72.97 b	103.95 a	80.76 b	5.04	0.013

Note: ALT: alanine aminotransferase. AST: aspartate aminotransferase. AKP: alkaline phosphatase. CAT: catalase. T-GSH: total glutathione. T-AOC: total antioxidant capacity. T-SOD: total superoxide dismutase. MDA: malondialdehyde. ROS: reactive oxygen species. Data in same row with different letters indicates significant differences (*p* < 0.05, n = 3).

**Table 4:** Effect of betaine on the lipid metabolism of largemouth bass.

Parameters	Diets			Pooled SEM	*p*-Value
	BD	LF	LFB4.0		ANOVA
Serum					
TG (mmol/L)	12.73 b	25.61 a	12.59 b	0.78	<0.001
NEFA (mmol/L)	69.90 b	82.95 a	71.67 b	1.13	<0.001
T-CHO (mmol/L)	11.30 c	35.03 a	14.63 b	0.66	<0.001

Note: TG: triglyceride. NEFA: nonesterified free fatty acids. T-CHO: total cholesterol. Data in same row with different letters indicates significant differences (*p* < 0.05, n = 3).

**Table 5:** Effect of betaine on the glucose metabolism of largemouth bass.

Parameters	Diets			Pooled SEM	*p*-Value
	BD	LF	LFB4.0		ANOVA
Liver					
Glycogen (mg/g)	34.78 c	58.67 a	44.26 b	2.01	0.001
Pyruvate (μmol/mg prot)	0.76 c	1.59 a	1.23 b	0.07	0.001
ATP (nmol/mg prot)	61.72 a	30.37 c	45.08 b	2.57	<0.001
CS (U/mg prot)	5.20 a	2.13 b	4.27 a	0.26	<0.001

Note: ATP: adenosine triphosphate. CS: citrate synthase. Data in same row with different letters indicates significant differences (*p* < 0.05, n = 3).

**Table 6:** Effects of betaine on BAs-related indicators of largemouth bass.

Parameters	Diets			Pooled SEM	*p*-Value
	BD	LF	LFB4.0		ANOVA
Liver					
Total BAs (ng/mg)	185.90 b	1682.13 a	308.12 b	82.59	<0.001
Total primary BAs (ng/mg)	184.59 b	1676.17 a	307.23 b	82.34	<0.001
Total secondary BAs (ng/mg)	1.31 ab	5.96 a	0.89 b	0.88	0.031
Total conjugated BAs (ng/mg)	183.59 b	1671.28 a	305.61 b	82.36	<0.001
Total unconjugated BAs (ng/mg)	2.31	10.85	2.51	1.72	0.052
Intestine content					
Total BAs (ng/mg)	10,006.04	5106.90	7253.80	1303.08	0.124
Total primary BAs (ng/mg)	9970.46	5100.84	7239.99	1300.91	0.125
Total secondary BAs (ng/mg)	35.58 a	6.06 b	13.82 b	2.29	0.001
Total conjugated BAs (ng/mg)	9980.59	5104.36	7251.18	1301.79	0.125
Total unconjugated BAs (ng/mg)	25.46 a	2.54 b	2.63 b	1.32	<0.001
Serum					
Total BAs (ng/mL)	780.29 b	2522.58 a	1385.29 b	165.94	0.004
Total primary BAs (ng/mL)	656.89 b	2368.18 a	1225.47 b	160.66	0.003
Total secondary BAs (ng/mL)	123.41	154.40	159.83	7.94	0.336
Total conjugated BAs (ng/mL)	659.25 b	2343.47 a	1251.71 b	169.70	0.005
Total unconjugated BAs (ng/mL)	121.05 b	179.11 a	133.59 ab	10.71	0.027
TBIL (μmol/L)	46.34 b	68.96 a	54.25 b	2.77	0.003
DBIL (μmol/L)	35.38 b	56.16 a	42.36 b	2.57	0.004
Gallbladder					
Total BAs (μmol/L)	346.50	304.44	305.96	19.04	0.316
Liver					
FXR (ng/g)	998.24 a	604.82 c	837.74 b	26.20	<0.001
SHP (ng/g)	500.16 a	290.62 c	349.66 b	9.28	<0.001
CYP7A1 (ng/g)	54.64 b	94.38 a	64.02 b	2.17	<0.001
CYP27A1 (ng/g)	290.43 b	419.42 a	358.10 ab	17.51	0.007
Intestine					
BSH (ng/g)	2808.46 a	1928.90 b	3329.32 a	137.71	0.001

Note: BAs: bile acids. TBIL: total bilirubin. DBIL: direct bilirubin. FXR: farnesoid-X-receptor. SHP: short heterodimer partner. CYP7A1: cytochrome P450 7A1. CYP27A1: cytochrome P450 27A1. BSH: bile salt hydrolase. Total primary BAs = Glycocholic acid (GCA) + Glycochenodeoxycholic acid (GCDCA) + Taurocholic acid (TCA) + Taurochenodeoxycholic acid (TCDCA) + Tauro-alpha-muricholic acid (T-α-MCA) + Tauro-beta-muricholic acid (T-β-MCA) + Taurohyocholic acid (THCA) + Cholic acid (CA) + Chenodeoxycholic acid (CDCA) + Hyocholic acid (HCA) + Norcholic acid (NorCA) + Glycohyocholic acid (GHCA) + Chenodeoxycholic acid-3-β-D-glucuronide (CDCA-3Gln) + 3-Dehydrocholic acid (3-DHCA) + Ursocholic acid (UCA) +12-ketochenodeoxycholicacid (12-KCDCA) + 3β-Cholic acid (3β-CA). Total secondary BAs = Allocholic Acid (ACA) + Ursodeoxycholic acid (UDCA) + Deoxycholic acid (DCA) + Tauroursodeoxycholic acid (TUDCA) + Hyodeoxycholic acid (HDCA) + Glycodeoxycholic acid (GDCA) + Glycolithocholic acid (GLCA) +7-ketoLithocholic acid (7-KLCA) + Taurohyodeoxycholic acid (THDCA) + Taurolithocholic acid (TLCA) + Taurodeoxycholate acid (TDCA) + Lithocholic acid (LCA) + isolithocholic acid (isoLCA) + 12-ketolithocholic acid (12-KLCA) + Dehydrolithocholic acid (DHLCA) + 7-ketodeoxycholic acid (7-KDCA) + Tauro-omega-muricholic acid (T-ω-MCA) + Lithocholic acid 3-sulfate (LCA-3S). Total conjugated BAs = GCA + GCDCA + TCA + TCDCA + TUDCA + GDCA + GLCA + T-α-MCA + T-β-MCA + THDCA + THCA + TLCA + TDCA + GHCA + CDCA-3Gln + T-ω-MCA. Total unconjugated BAs = ACA + DCA + UDCA + HDCA+ 7-KLCA + CA + LCA + CDCA + HCA + NorCA + isoLCA + 12-KLCA + DHLCA + 3-DHCA + UCA + 7-KDCA + 3β-CA + LCA-3S + 12-KCDCA. Data in same row with different letters indicates significant differences (*p* < 0.05, n = 3).

## Data Availability

All data supporting our findings are included in the manuscript.

## Supplementary Materials

The following supporting information can be downloaded at: https://www.mdpi.com/article/10.3390/antiox15080982/s1, Table S1: Sequences of primer used in qPCR. Table S2: Information on bile acid standards. Figure S1: Principal Component Analysis (PCA) of the metabolome of BAs. (A) The PCA of the liver. (B) The PCA of the intestinal content. (C) The PCA of the serum. References [13,40,41] are cited in the Supplementary Materials.

## Abbreviations

ATP: adenosine triphosphate; ALT: alanine aminotransferase; AST: aspartate aminotransferase; AFR: abdominal fat rate; ACA: allocholic Acid; BA: bile acid; Bsep: bile salt export pump; BSH: bile salt hydrolase; CPC: cottonseed concentrated protein; CYP7A1: cytochrome P450 7A1; CYP27A1: cytochrome P450 27A1; CS: citrate synthase; CF: condition factor; CAT: catalase; CDCA-3Gln: chenodeoxycholic acid-3-β-D-glucuronide; CDCA: chenodeoxycholic acid; CA: cholic acid; DBIL: direct bilirubin; DCA: deoxycholic acid; DHLCA: dehydrolithocholic acid; FM: fishmeal; FR: feeding rate; FCR: feed conversion ratio; FXR: farnesoid-X-receptor; GCA: glycocholic acid; GCDCA: glycochenodeoxycholic acid; GHCA: glycohyocholic acid; GDCA: glycodeoxycholic acid; GLCA: glycolithocholic acid; HSI: hepatosomatic index; HCA: hyocholic acid; HDCA: hyodeoxycholic acid; isoLCA: isolithocholic acid; LCA: lithocholic acid; LCA-3S: lithocholic acid 3-sulfate; MDA: malondialdehyde; NEFA: nonesterified free fatty acids; NorCA: norcholic acid; ROS: reactive oxygen species; SHP: short heterodimer partner; TBIL: total bilirubin; TG: triglyceride; T-CHO: total cholesterol; T-GSH: total glutathione; T-AOC: total antioxidant capacity; T-SOD: total superoxide dismutase; TCA: taurocholic acid; TCDCA: taurochenodeoxycholic acid; T-α-MCA: tauro-alpha-muricholic acid; T-β-MCA: tauro-beta-muricholic acid; THCA: taurohyocholic acid; TUDCA: tauroursodeoxycholic acid; THDCA: taurohyodeoxycholic acid; TLCA: taurolithocholic acid; TDCA: taurodeoxycholate acid; T-ω-MCA: tauro-omega-muricholic acid; UCA: ursocholic acid; UDCA: ursodeoxycholic acid; VSI: viscerosomatic index; WGR: wight gain rate; 3-DHCA: 3-dehydrocholic acid; 3β-CA: 3β-Cholic acid; 7-KLCA: 7-ketoLithocholic acid; 7-KDCA: 7-ketodeoxycholic acid; 12-KCDCA: 12-ketochenodeoxycholicacid; 12-KLCA: 12-ketolithocholic acid.
