# Supplementary material for: Enhancing Hepatic Antioxidant Capacity and Carbohydrate, Lipid, and Bile Acid Metabolism in Largemouth Bass Through Dietary Betaine Supplementation
Source: Antioxidants (Basel). 2026 Aug 7;15(8):982. doi: 10.3390/antiox15080982 (PMC13510068; doi:10.3390/antiox15080982)
Supplement: Supplementary file 1 [file antioxidants-15-00982-s001.zip › antioxidants-4443475-supplementary.pdf]

Table S1 Sequences of primer used in qPCR.

Gene	Forward primer (5′ to 3′)	Reverse primer (5′ to 3′)	Reference
β-actin	AAAGGGAAATCGTGCGTGAC	AAGGAAGGCTGGAAGAGGG	[41]
acc1	ATCCCTCTTTGCCACTGTTG	GAGGTGATGTTGCTCGCATA	[41]
fasn	TGTGGTGCTGAACTCTCTGG	CATGCCTAGTGGGGAGTTGT	[41]
srebp1	GAGGACACCAAGCCGAATG	TGCCAGAGGGTTGAGGGA	[40]
atgl	CCATGATGCTCCCCTACACT	GGCAGATACACTTCGGGAAA	[41]
pparα	CCACCGCAATGGTCGATATG	TGCTGTTGATGGACTGGGAAA	[13]
gk	ACAGAGTGGTGGACGAGACC	TCGTTCACCAGCTTCATCAG	[41]
pk	CCTATCGGAATTGCACTGGA	TTCTTGTAGTCGAGCCAGAG	[41]
g6pase	GGGAGTCCAGGTGTGTGTCT	CAGCGAAGGAGGTCAAGAAG	[41]
pepck	GGCAAAACCTGGAAGCAAGG	ATAATGGCGTCGATGGGGAC	[41]
besp	AGATGCTCCGTACCAAGCG	CACCAAGTAACCTCCAAACCTAT	[40]
ntcp	CAAGGCTGTCGGAGGCAACG	ATGGAGGAGAAGGGAACG	[40]
mrp4	GGGTCAGCTTAGCAAGAGCA	TCAAAGAGGTGCCTTCCCAC	XM_038720596.1

Table S2 Information on bile acid standards.

Name	Abbreviation	Manufacturer
Glycocholic acid	GCA	Shanghai ZZBIO, China
Glycochenodeoxycholic acid	GCDCA	Shanghai ZZBIO, China
Taurocholic acid	TCA	Shanghai ZZBIO, China
Taurochenodeoxycholic acid	TCDCA	Shanghai ZZBIO, China
Glycoursodeoxycholic acid	GUDCA	Shanghai ZZBIO, China
Allocholic acid	ACA	Toronto Research Chemicals
Ursodeoxycholic acid	UDCA	Sigma
Deoxycholic acid	DCA	Sigma
Tauroursodeoxycholic acid	TUDCA	Shanghai ZZBIO, China
Hyodeoxycholic acid	HDCA	Shanghai ZZBIO, China
Apocholic acid	Apo-CA	Shanghai ZZBIO, China
Glycodeoxycholic acid	GCDCA	Shanghai ZZBIO, China
Glycolithocholic acid	GLCA	Shanghai ZZBIO, China
Alpha muricholic acid	α-MCA	Shanghai ZZBIO, China
Beta- muricholic acid	β-MCA	Shanghai ZZBIO, China
7-keto-Lithocholic acid	7-KLCA	Shanghai ZZBIO, China
Tauro-α-muricholic acid	T-α-MCA	Shanghai ZZBIO, China
Tauro-β- muricholic acid	T-β-MCA	Shanghai ZZBIO, China
Omega-murichoclic acid	ω-MCA	Shanghai ZZBIO, China
Murideoxycholic acid	MDCA	Shanghai ZZBIO, China
Taurohyodeoxycholic acid	THDCA	Shanghai ZZBIO, China
Taurohyocholic acid	THCA	Shanghai ZZBIO, China
Taurolithocholic acid	TLCA	Shanghai ZZBIO, China
Taurochenodeoxycholic acid	TCDCA	Shanghai ZZBIO, China
Lithocholic acid	LCA	Sigma
Cholic acid	CA	Sigma
Chenodeoxycholic acid	CDCA	Sigma
Hyocholic acid	HCA	Shanghai ZZBIO, China
Norcholic acid	NorCA	Toronto Research Chemicals
Glycohyocholic acid	GHCA	Shanghai ZZBIO, China
23-Nordeoxycholic acid	23-NorDCA	Toronto Research Chemicals
isolithocholic acid	isoLCA	Shanghai ZZBIO, China
12-ketolithocholic acid	12-KLCA	Shanghai ZZBIO, China
Dehydrolithocholic acid	DHLCA	Shanghai ZZBIO, China
Lithocholic acid 3-sulfate	LCA-3S	Shanghai ZZBIO, China
3β-Ursodeoxycholic acid	3β-UDCA	Toronto Research Chemicals
Chenodeoxycholic acid 24-Acyl-beta-D-glucuronide	CDCA-24-Acyl-β-D-Glu	Toronto Research Chemicals
12-ketochenodeoxycholicacid	12-KCDCA	TLC Pharmaceutical Standards Ltd.
7,12-Diketolithocholic acid	7,12-DKLCA	Shanghai ZZBIO, China
Dehydrocholic acid	DHCA	Shanghai ZZBIO, China
Ursocholic acid	UCA	Toronto Research Chemicals
Isodeoxycholic acid	isoDCA	Shanghai ZZBIO, China

3β-cholic acid	3β-CA	Toronto Research Chemicals
Chenodeoxycholic acid-3-β-D-glucuronide	CDCA-3Gln	Toronto Research Chemicals
3-Dehydrocholic acid	3-DHCA	Shanghai ZZBIO, China
7-Ketodeoxycholic acid	7-KDCA	Shanghai ZZBIO, China
Tauro-omega-muricholic acid	T-ω-MCA	Shanghai ZZBIO, China

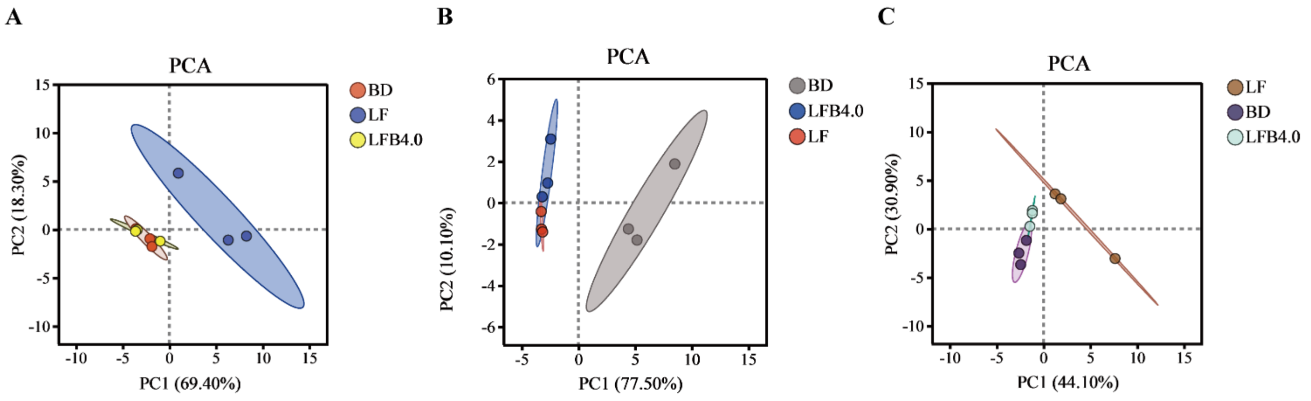

Fig. S1 Principal Component Analysis (PCA) of the metabolome of BAs. (A) The PCA of the liver. (B) The PCA of the intestinal content. (C) The PCA of the serum.
